# Supplementary material for: Riemannian L-systems: modelling growing forms in curved spaces
Source: Quant Plant Biol. 2025 Jul 1;6:e38. doi: 10.1017/qpb.2025.10014 (PMC12722102; doi:10.1017/qpb.2025.10014)
Supplement: Godin and Boudon supplementary material [file S2632882825100143sup001.zip › LegendsSupplementaryMovies (1).docx]

Supplementary movie #1: Fluid deformation of a fractal by substrate space growth.

Supplementary movie #2: Feedback of substrate space growth on form on a convected form

Supplementary movie #3: Growth of a kidney fern

Supplementary movie #4: Translation movement of a von-Koch flake in an abstract Riemannian space with metric increasing from a source point.

Supplementary movie #5.1: Effect of increasing gravitropism on a branching system.

Supplementary movie #5.2: Effect of a change of intensity of a source point-induced metric, and increasing with distance from source point.

Supplementary movie #5.3: Similar to Supplementary movie #5.2, with the source point located to the side of the branching system.
